# Statistical discovery of site inter-dependencies in sub-molecular hierarchical protein structuring

**DOI:** 10.1186/1687-4153-2012-8

**Published:** 2012-07-13

**Authors:** Kirk K Durston, David KY Chiu, Andrew KC Wong, Gary CL Li

**Affiliations:** 1 School of Computer Science, University of Guelph, 50 Stone Road East, Guelph, ON, N1G 2W1, Canada; 2 Department of System Design Engineering, University of Waterloo, 200 University Ave. W, Waterloo, ON, N2L 3G1, Canada

**Keywords:** *k*-modes algorithm, Site cluster, Associations, Ubiquitin, Transthyretin, Pattern discovery, Cluster tree, Attribute clustering, Protein structural sub-domains

## Abstract

**Background:**

Much progress has been made in understanding the 3D structure of proteins using methods such as NMR and X-ray crystallography. The resulting 3D structures are extremely informative, but do not always reveal which sites and residues within the structure are of special importance. Recently, there are indications that multiple-residue, sub-domain structural relationships within the larger 3D consensus structure of a protein can be inferred from the analysis of the multiple sequence alignment data of a protein family. These intra-dependent clusters of associated sites are used to indicate hierarchical inter-residue relationships within the 3D structure. To reveal the patterns of associations among individual amino acids or sub-domain components within the structure, we apply a *k*-modes attribute (aligned site) clustering algorithm to the ubiquitin and transthyretin families in order to discover associations among groups of sites within the multiple sequence alignment. We then observe what these associations imply within the 3D structure of these two protein families.

**Results:**

The *k*-modes site clustering algorithm we developed maximizes the intra-group interdependencies based on a normalized mutual information measure. The clusters formed correspond to sub-structural components or binding and interface locations. Applying this data-directed method to the ubiquitin and transthyretin protein family multiple sequence alignments as a test bed, we located numerous interesting associations of interdependent sites. These clusters were then arranged into cluster tree diagrams which revealed four structural sub-domains within the single domain structure of ubiquitin and a single large sub-domain within transthyretin associated with the interface among transthyretin monomers. In addition, several clusters of mutually interdependent sites were discovered for each protein family, each of which appear to play an important role in the molecular structure and/or function.

**Conclusions:**

Our results demonstrate that the method we present here using a *k-*modes site clustering algorithm based on interdependency evaluation among sites obtained from a sequence alignment of homologous proteins can provide significant insights into the complex, hierarchical inter-residue structural relationships within the 3D structure of a protein family.

## Introduction

The determination of protein 3D structure using methods such as NMR and X-ray crystallography has made tremendous progress. Although the 3D structure of many proteins has been solved, there still remains the problem of understanding the internal relationships within the structure. Certain residues may require specific associations with other residues within the structure that are not necessarily spatially proximal. Certain pairwise, third-order, fourth-order, and higher-order associations may be essential for obtaining a stable structure, while other parts of the structure have a less important role. The challenge is to be able to identify key structural associations within the larger structure, with the objective of understanding what role they play within the larger structure or global function of the protein.

Granular computing is emerging as a computing paradigm of information processing based on the abstraction of information entities called information granules [1-3], which we define here as related entities that are abstracted from the protein family multiple sequence alignment data based on a possible shared function. Functional bioinformatics is a sub-discipline of bioinformatics that incorporates functionality into the analysis of biopolymers [4-6]. Within a multiple sequence alignment, each column represents an aligned site, where a *site* refers to a location within an amino acid sequence*.* For example, a set of aligned sequences for a protein family that, on average, consists of 150 amino acid positions (with inserted gaps), would have 150 sites in its sequence. It is useful for estimating the functionality characteristics of sequence segments and/or functional sites, which can add enormous insights into understanding the characteristics of biopolymers.

The importance of patterns of associated sites within protein sequences has long been recognized. Structural and functional characteristics of a protein family may often be dependent upon two or more sites that maintain the stability of the molecule [7,8], such as in the situation of compensatory substitutions. In the late 1970s, based on statistics and information theory, Wong et al. [9] proposed a statistical analysis of site variability and interdependence in a protein family relating to the structural and functional relationships of sites in cytochrome c. Smith and Smith [10] developed a computer algorithm for detecting relationships among different sites in an amino acid sequence. Lichtarge et al. [11,12] have developed an evolutionary trace method (ETM) to identify clusters of sites associated with function, by mapping sites with a high degree of conservation onto the surface of the solved structure. Liu and Califano [13] have suggested a method for the functional classification of proteins through pattern discovery. Further work extended to data from aligned sequences has been conducted by Wong et al. [9,14,15] and Chiu et al. [16-18] in the developing of pattern discovery and analysis. An important goal of our proposed work is to extend our understanding of sub-molecular, internal relationships within the 3D structure of proteins by analyzing their multiple sequence alignments. In this article, we introduce a powerful new form of analysis based on the concept of granular computing and the *k*-modes attribute clustering algorithm (*k-*modes algorithm for abbreviation) to reveal statistical associations among multiple amino acids, using the aligned sequence data of both the ubiquitin and transthyretin protein families as the test bed. We make several discoveries, including three types of multiple amino acid associations as well as the observation that some of them form nested hierarchical branches and modules within the larger structure, indicating that our proposed granular computing method is conceptually sound and renders new understanding of internal relationships within the 3D structure of globular proteins.

## Application of the *k*-modes algorithm to multiple sequence alignments

Consider a given alignment of multiple sequences, possibly representing the different members of a protein or gene family. A multiple sequence alignment is defined formally as follows.

### Definition 1: (Multiple sequence alignment)

Consider that a family of molecular sequences is properly aligned. Let the aligned sites be represented as ***X*** = (*X*_1_, *X*_2_, …, *X*_*N*_) where *N* is the number of columns (aligned sites) in the multiple sequence alignment. Each aligned site is considered as an *attribute*. A realization of ***X*** is a particular sequence within the alignment and can be denoted as ***x***_*i*_ = (*x*_*i*1_, *x*_*i*2_,…, *x*_*iN*_) where *i* is the row number within the alignment and *x*_*ij*_ may assume any value in its alphabet set *G*. For proteins, *G* consists of the 20 amino acids and for DNA or RNA, *G* consists of the 4 nucleotides. We refer to an ensemble of outcomes of *X* as a *Multiple Sequence Alignment*

To clarify, a multiple sequence alignment consisting of *M* sequences and *N* columns (aligned sites or attributes) is shown below.

$$\begin{array}{l} X_{1},X_{2},X_{3}\text{,}\ldots ,X_{N}\\ x_{11},x_{12},x_{13}\text{,}\ldots x_{1N}\\ x_{21},x_{22},x_{23}\text{,}\ldots x_{2N}\\ .\\ .\\ .\\ x_{\text{M}1},x_{\text{M}2},x_{\text{M}3}\text{,}\ldots x_{\mathrm{MN}} \end{array}$$

where *Xn* represents the column/aligned site/attribute number and *x*_*ij*_ represents the particular amino acid or nucleotide found in row *i* and column *j*. For a protein family, the data for the two-dimensional array is the multiple sequence alignment for the family contained in databases such as Pfam [19,20].

## Evaluating interdependency between attributes

For aligned protein sequences, an aligned site is considered as an attribute. To evaluate the interdependency between two aligned sites, we use the method proposed by Wong et al. [9,21] where the interdependency redundancy measure between two attributes *X*_*i*_ and *X*_*i'*_ is given by the normalized mutual information

(1) $$R_{ii^{\prime }}=\frac{I\left(X_{i},X_{i^{\prime }}\right)}{H\left(X_{i},X_{i^{\prime }}\right)}$$

where

(2) $$I\left(X_{i},X_{i^{\prime }}\right)=\sum_{x\in X_{i}}\sum_{y\in X_{i^{\prime }}}p\left(x,y\right)log\left(\frac{p\left(x,y\right)}{p\left(x\right)p\left(y\right)}\right)$$

is the mutual information between *X*_*i*_ and *X*_*i'*_ (a measure of the average decrease in uncertainty about *X*_*i*_ that results from learning the value of *X*_*i'*_) and

(3) $$H\left(X_{i},X_{i^{\prime }}\right)=-\sum_{x\in X_{i}}\sum_{y\in X_{i^{\prime }}}p\left(x,y\right)logp\left(x,y\right)$$

is the joint entropy between *X*_*i*_ and *X*_*i'*_. Since *I*(*X*_*i*,_*X*_*i'*_) increases with the number of possible attribute values, *R*_*ii'*_ must be normalized. This avoids biasing the search for associations among sites toward larger clusters, which may actually have a low level of internal interdependency. The function *p* refers to the estimated probability from the sample data.

One should note that the stronger the interdependency between *X*_*i*_ and *X*_*i'*_, the higher the *I*(*X*_*i*_, *X*_*i'*_) value. As indicated by Equation (2), from the statistics standpoint, the normalized *R*_*ii'*_ also accounts for the amount of deviation from the independence hypothesis between *X*_*i*_ and *X*_*i'*_. *R*_*ii'*_ = 1 if *X*_*i*_ and *X*_*i'*_ are perfectly dependent and *R*_*ii'*_ = 0 if they are completely independent. Since *R*_*ii'*_ is a normalized interdependency measure, the interdependence relationship is not affected by the number of attribute values. If there is an interdependency between two attributes, there is a greater degree of correlation between them than when compared to two attributes that are less interdependent or independent. For this reason, *R*_*ii'*_ is able to measure the interdependence or correlation between attributes [9,15]. If *R*_*ii'*_ > *R*_*ih*_, *h ∈*$\left\{1,\ldots ,N\right\}$, *h ≠ i* ≠ *i*′, the dependency between *X*_*i*_ and *X*_*i'*_ is greater than that between *X*_*i*_ and *X*_*h*_. In searching for higher-order relations between attributes in a sequence alignment, we use *R*_*ii'*_ to measure the interdependence between attributes *X*_*i*_ and *X*_*i'*_. On the basis of *R*_*ii'*_, a statistical test is introduced to test whether or not two attributes are interdependent (or are deviating from independence) [22]. Recall that 0 ≤ *R*_*ii'*_ ≤ 1, and *R*_*ii'*_ = 0 if *X*_*i*_ and *X*_*i'*_ are totally independent, and *R*_*ii'*_ = 1 if totally dependent. The test, in terms of *R*_*ii'*_, then becomes as follows.

Two attributes *X*_*i*_ and *X*_*i'*_ are dependent if *R*_*ii'*_ ≥ *c*^2^_(*Gi*–1)(*Gi'*–1)_/2*n H*(*X*_*i*_, *X*_*i'*_) and false otherwise, where *c*^2^_(*Gi*–1)(*Gi'*–1)_ represents a chi-square distribution with (*Gi*–1)(*Gi'*–1) degrees of freedom (recall that for proteins *G* represents the alphabet set of 20 amino acids) and *n* represents the number of specimens in the cluster.

For sequences that presume to encode embedded functionality such as DNA, RNA, or proteins, *I*(*X*_*i*_, *X*_*i'*_) can be considered to reflect the mutual *functional* information governed by known (or predicted) functionality of a pair of sites. A measure of functional complexity (FC) was proposed by Durston et al. [6] for the case where the sequences are specified by a defined input class. Here, however, we are measuring the FC of the relationships *between* aligned sites, as specified by the attribute cluster.

To estimate the overall significant dependence of an attribute *X*_*i*_ with other attributes in a cluster of sites, the sum of normalized interdependency redundancy *SR*(*i*) of *X*_*i*_ with other attributes in the data array is calculated, where

(4) $$SR\left(i\right)=\sum_{(i,i^{\prime })\in N^{*}}R_{i,i^{\prime }}$$

and N* is the set of (*i,i'*) attribute pairs [9,15,17,21].

### Definition 2: (Mode of a set of attributes)

Within a cluster of attributes, we define the *mode* as the attribute with the highest normalized interdependency redundancy (*SR*) value. Formally, *X*_*i*_ is the mode of cluster *X* if

*SR*(*i*) ≥ *SR*(*i'*) for all attributes *X*_*i'*_ in *X.*

For the purpose of this article, and to compare clusters of different numbers of attributes, the highest normalized interdependency redundancy (*SR*(*i*)) divided by the number of attribute pairs N* in the cluster (i.e., *SR*(*i*)/N*) will be designated as *SR*(*mode*). The *SR*(*mode*), therefore, in quantifying the interdependency between the mode and the other attributes within the cluster, provides one method to quantify the average degree of interdependency within the cluster, as well as an objective method to rank different attribute clusters in terms of their internal interdependency.

Often, within a sequence alignment, it may be that two or more sites are closely associated by their amino acid composition, possibly due to residue–residue contacts [7]. It has been found that such amino acid associations could form compensatory relationships in certain mutational events [7]. As a result, amino acids at one site could have a functional relationship with particular amino acids at the other sites within the associated sites. We define these amino acid associations as *patterns* if the association is statistically significant.

### Definition 3: (pattern)

An *event* is an observation of an instance in a given data ensemble, either involving a single value or multiple jointly observed values. A *single-valued event* is referred to as a *primary event* and is a realization of *X*_*i*_ that takes on a value from $\alpha_{i}$, where *α* represents a symbol from the alphabet set *G*. For a primary event, only one outcome of a variable in *X* is involved. A multiple valued event is referred to as a *compound event*. A pattern is a compound event (an observation from a subset of *n* different variables of the *N*-tuple ***X***) which is statistically significant as reflected by its frequency of occurrences [23,24]. A pattern is denoted as $\lambda =\left(\alpha_{i}^{p},\alpha_{i^{\prime }}^{q},\ldots ,\alpha_{n}^{r}\right)$ where (*i*, *i'*, *i'',* …, *n*) is a set of aligned site indices (e.g., sites 2, 15, and 19), within the multiple sequence alignment. It represents the joint outcomes of $X_{i}=\alpha_{i}^{p}$, $X_{i^{\prime }}=\alpha_{i^{\prime }}^{q}$, …, $X_{n}=\alpha_{n}^{r}$ and that such association is statistically significant in the sense that the frequency of their occurrences deviate significantly from the default expected frequency if they are just a random occurrence or if they are a completely independent set.

For example, cluster (11) in Table 1 has two third-order patterns (sites 60, 63–64), where λ_1_ = amino acid pattern NKE, and λ_2_ = amino acid pattern GDG, in the corresponding sites in the alignment. Here, both patterns on these three sites are statistically significant according to their frequency of occurrences as observed in the sub-array of the aligned protein data.

**Table 1:** Attribute clusters with two high residual patterns

**Cluster**	**Sites**	***k***	***λ***_**1**_**amino acids**	***λ***_**1**_**-adjusted residual**	***λ***_**2**_**amino acids**	***λ***_**2**_**-adjusted residual**
10	54–59	6	RTLSDY	31.0	RTLADY	30.5
11	60,63–64	3	NKE	29.9	GDG	14.3
3	18–19,21	3	ESD	13.4	EPD	9.6

## Attribute clustering

In traditional pattern recognition, clustering is a process that groups similar samples together, maximizing the intra-group similarity and inter-group dissimilarity based on certain similarity or distance measures. An example is the method proposed by Li and Scheraga [25] for grouping similar sequences on the basis of their *information distance*, the length of the smallest binary computer program that will convert two sequences to each other. Clustering algorithms that group according to similarity between attributes are not appropriate for clustering in terms of interdependency, since dissimilar attributes may be interdependent. (For example, two aligned sites may be composed of dissimilar amino acids, yet be interdependent.)

If we analyze the relationship among attributes with the objective of clustering them into groups, we consider their interdependence instead. Using a sample clustering analogy in clustering attributes, we should maximize the intra-group interdependence (correlation) and inter-group independence. Hence, the attribute clusters within the sequence alignment could be obtained by using attribute interdependence values for associations.

There is a subtle difference between attribute interdependence and statistically significant patterns. The former describes dependence between attributes such as sites within an amino acid sequence, while the latter relates an associated set of amino acids at a respective set of sites in a sequence as joint events based on their statistically significant association. (To clarify, taking the same example 11 in Table 1, we observe that three attributes are mutually dependent. Yet, within these three sites we observe two third-order events λ_1_ = NKE and λ_2_ = GDG which are statistically interdependent.) Hence, one third-order cluster of aligned sites can contain two or more third-order patterns of amino acids within those sites. We could anticipate that attribute clusters usually contain statistically significant patterns among the amino acids in the clusters. Before we define attribute clusters, we first introduce the algorithm by which attribute clusters could be found.

## Attribute clustering algorithm

For sample clustering, a well-known process is the *kmedoids* algorithm [26]. To cluster samples into *k* groups, it first selects a random sample to represent each group and considers it as the center or *medoid* for that group. Thus, it selects *k* samples around which to build *k* groups. That is why the algorithm is referred to as the *k-medoids* algorithm. Using a distance measure, it clusters each of the remaining samples into the group for which the sample is closest to the medoid. After the first round, it updates a sample as the medoid of a group if the sum of distances of that sample to all other samples in that group is minimal. Based on their closest distance to these new medoids, all samples are then regrouped. The process is repeated until there is no more shift of samples between clusters. This method can work well for similarity-based clustering, but as pointed out earlier, interdependency might not be a function of similarity; two very dissimilar attributes may actually be interdependent. That is, different amino acids at distant sites could still be inter-associated if their occurrences are observed together.

The ETM, mentioned earlier, forms clusters of highly conserved sites that, when mapped to a 3D model, are correlated with a function such as binding. In this approach, an interdependency measure is not used. Instead, interdependency is inferred. Also, the interdependency that is inferred is between the function and the cluster of highly conserved sites. The sites (or attributes) may not necessarily all be interdependent between themselves. Furthermore, the ETM can be used only in proteins that have a known structure and are relatively free of noise.

**Figure 1:**
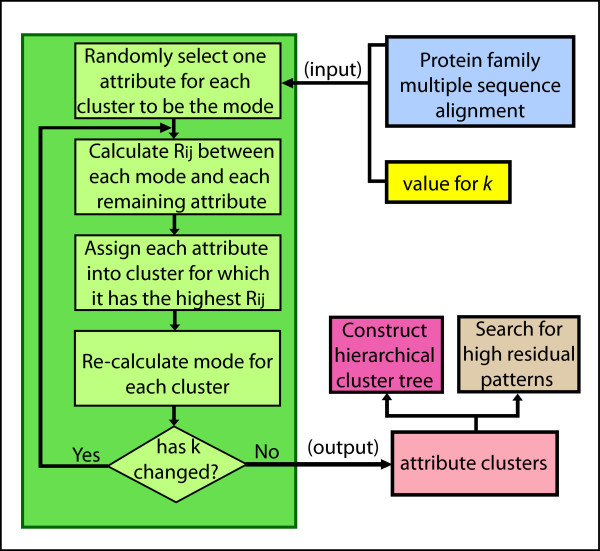
**Overview of algorithm for attribute clustering and pattern discovery.** The *k*-modes algorithm is summarized in the green portion of the flowchart. The algorithm can be run for a particular value for *k* or for multiple values. In order to build a hierarchical cluster tree for a protein family, represented by a multiple sequence alignment with *N* aligned sites, the value of *k* is increased each time from a starting value of *N* – 1 down to 2. The entire set of attribute clusters can then be arranged into a cluster tree for that protein family, or individual clusters can be computationally analyzed for patterns.

A concept similar to the *k-medoids* algorithm was developed by Wong and Wang [27] and adopted to cluster genes from micro-array gene expression data [15]. It is known as a *k*-modes attribute clustering algorithm. In the *k*-modes algorithm, we replace the medoid, representing the center of a cluster of samples in the *k*-medoids algorithm, with the ‘mode’ which is the attribute that has the highest *SR*(*i*) within that attribute cluster. The *k-*modes algorithm uses the interdependency redundancy measure *R*_*ii'*_ between attributes for attribute clustering instead of the similarity measure used in sample clustering. The *k-*modes algorithm, shown in Figure 1, then proceeds as follows.

The first step is initialization where *k*, the prescribed number of clusters, is inputted and an attribute is randomly selected for each cluster, representing a candidate for the mode of that cluster. Step 2 is to calculate the interdependency redundancy measure *R*_*ii'*_ between each cluster mode and each of the remaining attributes. Each of these remaining attributes is then assigned to the cluster for which it has the highest *R*_*ii'*_ value. Step 3 is to compute the updated mode for each of the *k* clusters (recalling that the initial mode was only a candidate used to start the process). In the subsequent iterative step, once the new mode of each of the *k* clusters has been calculated, steps 2 and 3 are repeated until the calculated mode for each of the *k* clusters does not change, that is, there is no exchange of attributes between attribute clusters. The algorithm is terminated at that point or, alternatively, at a pre-specified number of iterations.

For protein family multiple sequence alignments, all unassociated insertions and gaps are computationally removed from the previous procedure. Because it is generally recognized that sequence determines structure [28], and structure is important for function, it is assumed that each of the remaining sites within the sequence is either directly or indirectly interdependent with every other site in the sequence. To clarify, one pairwise interdependency may not be directly related to another attribute cluster consisting of three interdependent sites, but both clusters may be nested within larger clusters that are directly related to form a still larger cluster. An exception might be a cluster of sites that are interdependent only due to an external function, such as a highly conserved binding site. Thus, in order to discover the full range of the nested clusters, the *k*-modes algorithm is run for all values of *k* from *k* = *N –* 1 down to *k* = 2. For example, a multiple sequence alignment with 100 aligned sites would first be run with *k* set to *N –* 1 = 99. This would result in 1 pairwise cluster and 98 single attribute clusters to account for all 100 aligned sites. The value for *k* would be reduced by 1 each time all the way down to 2, which would yield 2 large attribute clusters comprising all 100 aligned sites. It is possible that the structure of a protein may not actually fit into two high-order attribute clusters. For example, a protein family that has a 3D structure composed of three structural domains may yield valid results for a *k* as small as 3 but not for a *k* as small as 2. If this is the case, it will become evident when the cluster tree of nested hierarchical attribute clusters is built, which will be explained shortly. Because the *k*-modes algorithm clustering method is data driven, it should yield the same results for multiple runs of the same *k*, even though a random attribute is chosen at the initial step. This was found to be the case for both protein families analyzed.

Regarding the complexity of the algorithm, the *k-*modes algorithm for *N* attributes and *n* samples requires *O*(*nN*) operations to assign each attribute to a cluster (step 2) and *O*(*nN*^2^) operations to compute the mode *X*_*m*_ for each cluster. If *t* represents the number of iterations, then the computational complexity of the *k-*modes algorithm is

(5) $$O\left[k\left(nN+nN^{2}\right)t\right]$$

or

(6) $$O\left(knN^{2}t\right)$$

This order of computational complexity is manageable within a reasonable space and time on any recent laptop computer.

An attribute cluster is formally defined as follows.

### Definition 4: (attribute cluster)

For an ensemble of data ***X*** = (*X*_1_, *X*_2_, …, *X*_*N*_) with *N* attributes, if *X* is partitioned vertically into *k* sub-arrays by the *k*-modes attribute clustering algorithm, then each sub-array is defined as an attribute cluster in the *k*-cluster configuration of ***X.***

Applying this definition to a protein sequence alignment, an attribute cluster is simply a cluster of sites that are mutually interdependent. For example, several attribute clusters for the ubiquitin family are shown in Table 2. One such attribute cluster is 11, consisting of sites 60, 63, and 64 in the aligned sequences.

**Table 2:** Representative clusters from each primary branch in ubiquitin

**Cluster**	**Sites**	**Highest pattern residual**	***k***	**Structural relationship**	**Functional factors**
1	7,9–11	25.9	4	Double bond between 7 and the other sites	11 is a binding site
2	12,14–17,20	57.6	6		12 is adjacent to binding site 11
3	18–19,21	13.4	3	Double bond between 18 and 21	
4	23,25–27	6.7	4	Double bond between 23 and other sites	27 is a binding site
5	29–31,41	22.6	4	30 in Van der Waals contact with 41	29 is a binding site
6	34–36,38	27.9	4	Van der Waals contact 34 & 36	34 is adjacent to binding site 33
7	37,39–40,43	20.8	4	37 bonds to 40	43 is adjacent to possible external recognition site 42 and external contact site 44
8	44–47	646.1	4	Van der Waals contact 44 & 47	sites 44 and 47 are external contact points, 47 is adjacent to major binding site 48
9	49–50,52	7.5	3		site 49 is an external contact point
10	54–59	31.0	6	8 internal H-bonds	
11	60,63–64	29.9	3		63 is a major binding site
12	66,68–70	27.8	4		site 70 is an external contact point
13	43,69,74	13.0	3	43 in tight Van der Waals contact with 69	74 is a possible external recognition site
14	70,72–73	22.6	3		70 is an external contact point, 73 is an external recognition site

In this study, we consider only those patterns observed within an attribute cluster even though some might span across attribute clusters with weak interdependence. The set of attributes (as random variables) within an attribute cluster is then defined as a new pattern subspace as below.

### Definition 5: (A significant pattern subspace)

Consider a subset ***Y*** of N* (N ≥ N* > 1) attributes of ***X*** such that at least one pattern is spanning that subset. Let $G_{y}=\left\{\lambda^{1},\lambda^{2},\ldots \lambda^{m}\right\}$ denote the set of *m* patterns contained in the subset ***Y***. We refer the vector subspace ***Y*** as a significant pattern subspace containing the *m* patterns which span that subspace.

For example, in Table 2, cluster 11 forms a transformed attribute, consisting of the attribute set of aligned sites 60, 63, and 64. The attribute cluster 11 is not the pattern, rather, it is a result contributed by two amino acid patterns λ_1_ = NKE and λ_2_ = GDG at two different subsequent instances in the significant pattern subspace (second row in Table 1). Note that the variables in a subset of ***X*** forming a subset ***Y*** in the new significant pattern subspace will not be overlapped, as our attribute clustering algorithm will ensure that they are disjoint. Later, we will discuss how a hierarchical structure can be constructed in the form of a cluster tree.

## Attribute clusters and information granules

The concept of granular computing allows analysis of granular units, in terms of modularity and hierarchy as a web structure of relationships [29-36]. Applying this concept to proteins entails analyzing them in terms of a hierarchical assembly of modules, each of which is associated with an attribute cluster.

Within a group composed of different types, a granule was originally conceptualized and served as a focal point of similar types within the group to study their collective properties [2]. For our problem of understanding the internal association relationships of a protein, the clusters of sites in the alignment can be considered as a set of granular units (which we called *information granules*) such that their information reflects the inferred associative properties of the protein family under study. A low-order attribute cluster may represent two or three residues that are in contact through H-bonds or in van der Waals interaction distances. The two or three residues can also be more distant from each other, yet be mutually associated, possibly through the effects the members of the cluster have on torsion angles and free energy during folding. Low-order clusters may be nested within higher-order clusters, which represent important sub-domain structural components.

**Figure 2:**
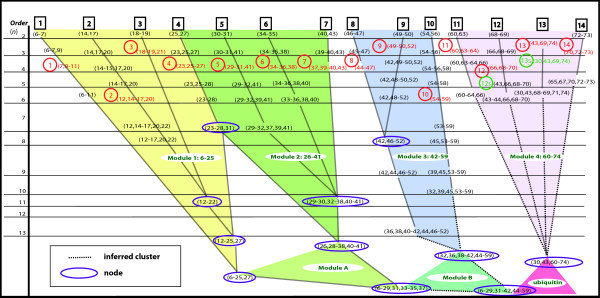
**Cluster tree for ubiquitin.** The attribute clusters discovered from the aligned sequence data for the ubiquitin family are shown above and organized vertically according to their order (the number of interdependent sites they contain). The organized clusters form primary branches, numbered 1 to 14 across the top of the figure. In each branch, the attribute cluster with the highest internal interdependency (highest *SR*(*mode*) value) was chosen as the representative cluster for that branch and is labeled according to its branch number. Two secondary clusters, discussed in the text, are labeled 12 s and 13 s. From this cluster tree, new insights can be gained into details of folding and structure.

To analyze the nested hierarchical relationships between the clusters, a cluster tree can be built, as illustrated in Figure 2. The cluster tree will also clearly distinguish between valid and invalid clusters that were forced by using a *k* value (the number of clusters) that was too small. The cluster tree can either be built computationally or manually. The first step is to arrange all the second order (pairwise, involving two attributes) clusters from left to right, according to approximately where the pairwise clusters appear in the sequence. (For example, a pairwise cluster composed of sites 1 and 3 would be on the left and the highest site number pairwise cluster would be on the right, with all others arranged in between.) Not all aligned sites will necessarily form pairwise clusters. The next step continues with all the third-order clusters (or clusters composed of three interdependent sites) and so on. If a second-order cluster is nested within a third-order cluster, then the third-order cluster should be placed directly beneath the second-order cluster to form the beginnings of one branch of the cluster tree. Most clusters will likely be nested within larger order clusters, so the lower-to-higher order nested clusters will form the branches of the cluster tree. Since some sites may be associated with more than one cluster, they may switch branches as the order of the clusters is increased. A *node* occurs when two or more branches converge to form a large cluster. Some nodes may be nested within even larger clusters forming a larger branch representing a larger portion of the protein. In this case, the entire section of branches that converges into a larger node comprising a significant portion of the protein is called a *module*. If a branch reaches a point where the cluster is not nested in a higher-order cluster, then that branch should be left unattached to the cluster tree; it may have functional or structural significance on a local scale and should be retained in the cluster tree diagram.

As already mentioned, each higher-order cluster should contain most of the sites included in the lower-order cluster(s) that are nested within it by choosing a reasonable number of clusters, or a *k* value that is not too small. The completed tree should be a valuable tool in identifying and understanding sub-molecular relationships, both functional and structural, within the generalized 3D structure of the protein family being investigated.

Each information granule is defined as an attribute cluster and allows for local analysis, as well as a global analysis under this framework. The hierarchical levels of analyses then provide multiple views into the protein structure including key residue–residue contacts, pairwise, third- and fourth-order relationships with multiple sites on up to larger sub-domains.

Table 2 lists 14 attribute clusters within the aligned sequences of the ubiquitin family. Each of these reflects complex variability and association information that can be analyzed and compared to the functional and structural characteristics necessary for the ubiquitin family. By studying these generated granules, detailed insights can be gained on the sub-molecular characteristics of the ubiquitin family.

To summarize our approach, Figure 1 shows a flow chart diagram for the attribute cluster analysis of protein families. The analysis begins with the downloading of a sequence alignment of all sequences predicted for a protein family. Redundant sequences are then removed so that the resulting alignment consists only of unique sequences for that family. The resulting alignment is then analyzed using our method to detect attribute clusters. The attribute clusters are then grouped into hierarchical families of clusters forming branches, some of which may form a hierarchical cluster tree, an example of which can be seen in Figure 2. The clusters are then analyzed for structural and functional contributions with the objective of better understanding the key internal hierarchical structural sub-domain relationships and at various levels.

### Statistical significance of results

Our approach is to locate statistically significant associations among sites in a multiple sequence alignment, as well as statistically significant patterns of amino acids within those associations. For locating associations between sites, the normalized mutual information *R*_*ii'*_ (Equation 1) between attributes *X*_*i*_ and *X*_*i'*_, known as redundancy, is used.

To ensure that patterns are statistically significant, the adjusted residual of each possible pattern within cluster is calculated [23]. The *residual* of a pattern λ is defined as the difference between the actual occurrence of λ and its expected occurrence, or

(7) δλ=oλ−eλ

where *eλ* is the expected occurrence of the pattern which, in this case, is that there are no patterns. The *adjusted residual* of the pattern λ is defined as

(8) $$\gamma \lambda =\frac{\delta \lambda }{\sqrt{\nu \lambda }}$$

where *νλ* is the variance of *δλ*. To find an amino acid pattern within a given cluster, a lower cutoff value for the adjusted residual is chosen. The adjusted residuals are calculated based on the amino acids observed within the multiple sequence alignment within the particular cluster being examined (i.e., only one cluster is analyzed at a time, which reduces the computational search to just the aligned sites in the cluster). An adjusted residual that is less than the cutoff value is rejected and an adjusted residual higher than the cutoff value is retained as significant to a certain confidence level. (For example, an adjusted residual of 2.58 means that an amino acid pattern is statistically significant to a confidence level of 99%, and an adjusted residual of 3.29 is statistically significant to a confidence level of 99.9%.)

In this way, only statistically significant results are obtained and spurious results preempted. Thus, statistical significance is the operative criterion throughout the search process, ensuring statistically significant results. For a multiple sequence alignment, the minimum number of non-redundant aligned sequences required for statistically significant results is five [23]. However, only very strong associations and patterns will be revealed with such a small sample size. Adding additional non-redundant sequences to the alignment will increase the likelihood of finding additional patterns within the same association of sites, and of locating the smaller associations among sites that may be nested within the stronger, larger associations. Thus, the larger the non-redundant multiple sequence alignment, the more attribute clustering detail can be resolved.

## Computational results for ubiquitin and transthyretin

Several dozen attribute clusters were generated by the program from the sequence alignment for both the ubiquitin and transthyretin (TTR) families, ranging from simple, pair-wise clusters up to one 58th-order cluster in the case of TTR. For clarity and logical flow, we shall discuss the results in the step-wise order as they emerged in the process of our analysis, dealing first with ubiquitin, then with TTR. To assist in clarity, a summary of the most significant results and predictions is shown in Table 3.

**Table 3:** Overview of experimental results and predictions

	**Results**	**Predictions for further research**
1.	Attribute clusters can be grouped into a hierarchical cluster tree composed of branches, nodes, and modules	Cluster trees may reveal details of folding constraints and other functionality relationships
2.	Within modules, two types of branches were found: (i) Type I; independent/non-interlacing with other branches and (ii) Type II; interlacing with other branches	The relationship between Types I and II branches may indicate constraints in the folding, tertiary structure and functionality of protein molecules
3.	Attribute clusters with highest *SR*(*mode*) values were most commonly third- and fourth-order (3 to 4 associated sites)	Support for the next largest building block in proteins above single amino acids is typically a 3 to 4 amino acid structural unit
4.	Three types of attribute clusters were found: (i) H-bonded clusters, (ii) Van der Waals clusters, and (iii) extended clusters	Identifying attribute clusters may support possibility of predicting protein tertiary structural relationships involving H-bonds, van der Waals interactions, or multiple site effects
5.	Representative clusters found in ubiquitin confirm that the statistical criterion used can identify structural constraints such as bonding, binding and recognition sites (Table 2)	Can be used to locate key sub-molecular components of a protein, as well as components critical for the function of that protein
6.	Ubiquitin molecule found to be composed of four major modules	Consistent with the four major areas of chemical shift perturbations between Ub_1_ and Ub_2_

## Ubiquitin

### The cluster tree

A cluster tree was assembled as described in the Section “Attribute clusters and information granules”, and is shown in Figure 2. Two types of branches can be observed. A Type I branch is made up of aligned site clusters which exhibit little or no interlacing with clusters residing in other branches (for example, primary branch (1) in Figure 2). The lack of interlacing between a Type 1 branch and other branches may indicate that the residues within a Type I branch are assembled completely prior to folding into the larger structure. The Type II branch, as illustrated by branches (5), (6), and (7), is made up of attribute clusters containing sites that interlace with sites in other branches. The overlapping/interlacing sites in the attribute clusters among Type II branches suggest that there is a strong structural relationship among those branches, giving robust structural stability in the region containing them. A prediction that arises out of the observation of two neighboring, strongly interlaced Type II branches, is that during translation, the two branches may fold into each other before folding as a cohesive unit back onto the part of the protein that has already been formed by earlier branches.

Figure 2 reveals that the 14 primary branches converged into 4 primary modules (labeled in Figure 2 as modules 1–4). Modules formed as the result of merging two or more primary modules are labeled as modules A and B (Figure 2). For example, modules 1 and 2 converged to form module A. Since branches 8, 9, and 10 were strongly interlaced, and showed no signs of converging with branches 11–14, branches 8–10 were grouped to form their own module 3. The strong interlacing of branches 11–14 resulted in the formation of module 4. Once the cluster tree was completed, the next steps were to analyze a representative cluster from each branch, and then analyze the four modules. The next three sections contain the results of these steps.

### Representative clusters

For the next step, we chose a set of representative clusters for detailed examination. Any cluster can be chosen to represent a primary branch, but for ubiquitin, the cluster with the highest *SR*(*mode*) value was chosen to be the representative cluster. The representative cluster for each branch is shown highlighted in red in Figure 2 and labeled 1 to 14, where the numeral denotes the primary branch number. Table 2 contains a list of the representative clusters for the primary branches 1–14 together with the highest adjusted residue value of the strongest pattern within each representative cluster.

Using the secondary structure revealed in the X-ray diffraction 1.8 angstrom solved structure 1UBQ [37,38] available from the Protein Data Bank, of the representative attribute clusters in each branch, we observe that (a) 11 out of 14 clusters included loops or turns; (b) 9 out of 14 included beta strands; and (c) 5 out of 14 included helices. We also note that three of the clusters occurred entirely within loops or turns, with only one cluster occurring entirely within a helix and only one entirely within a beta strand. Since loops and turns can be highly constrained structurally, it may explain why a higher occurrence of clusters included loops and turns.

Another observation from the representative clusters (highlighted in red in Figure 2) is the percentage of fourth-order (involving four sites) clusters. Of the set of clusters, one from each primary branch, that had the highest *SR*(*mode*) value (i.e., the strongest average interdependency between sites), 7 were fourth-order (50%), 5 were third-order (36%), sixth-order clusters comprised only 14%, and there were no fifth-order high *SR*(*mode*) value clusters in the individual branches. This suggests that the most common structural units within proteins may involve 3 to 4 amino acids. The node for Module 1 composed of the 21st-order cluster (6–25, 27) had a high *SR*(*mode*) value of 0.202. This high internal interdependency may be a strong predictor of a structural sub-domain. A high *SR*(*mode*) value at the node of a module indicates the level of internal interdependency and stability once the folding is complete for that module.

The *k*-modes algorithm found one or more patterns for each of the representative clusters. Recall that a pattern consists of an association of specific amino acids among the sites in the cluster. For statistical significance, an adjusted residual value was calculated for each pattern in a cluster. The residual value for each of the representative clusters shown in Table 2 is only for the pattern with the highest residual found within each representative cluster. Many of the clusters had other patterns as well. For example, two patterns for each of three different representative clusters are shown in Table 1. The fact that patterns were discovered for each of the representative clusters is verification that the attributes clusters discovered by this method contain high residual associations of amino acids.

### Classifying the clusters

**Figure 3:**
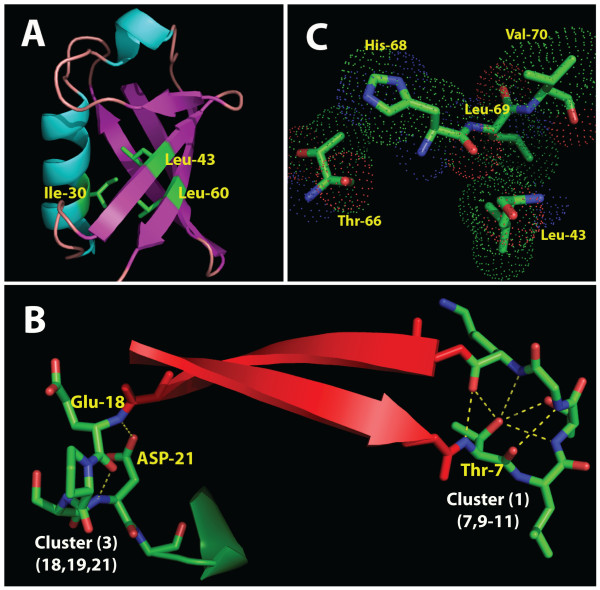
**Ubiquitin. (A)** The 3D structure of ubiquitin, using the 1UBQ solved structure. Part of cluster 13 s is also shown within the ubiquitin molecule. The three sites are all within van der Waals interaction distance of each other and may have an important role in the stability of the overall structure. **(B)** Module 1. This module contains two H-bonded clusters, which may play a role both in folding and then maintaining structural stability once folding is complete, especially cluster 1 with six H-bonds anchoring the loop. **(C)** Cluster 12 s, a strong example of a van der Waals cluster discovered by the *k*-modes algorithm.

The third step was to examine and classify the clusters to see what significance they had within the 3D structure. We note that the representative clusters chosen for their high *SR*(*mode*) values within each branch can be interpreted as functionally significant either for structure or external binding and interaction. The 3D structure of ubiquitin is shown in Figure 3A, using the solved structure 1UBQ. In Table 2, comparisons between the clusters and model 1UBQ were made. For functional significance, known and suspected sites that are involved in external binding or recognition are as follows:

(a)Sites 6, 11, 27, 29, 33, 48, and 63 are thought to be possible binding sites in the formation of poly-ubiquitin, with sites 48 and 63 thought to be the most common [39,40].

(b)Sites 8, 44, and 70 form hydrophobic patches that appear to contact the corresponding sites in Ub_2_, stabilizing the dimer in its closed conformation [41].

(c)Sites 47, 49, and 71 form hydrogen bonds with corresponding sites in the other moiety in Lys-48 linked Ub_2_[42].

(d)Sites 42, 72, and 74 are located at the interface between the two ubiquitin domains, which may represent possible interaction sites that distinguish multi-ubiquitin chains from a single ubiquitin molecule, providing a means of external recognition [42].

Using these comparisons, the representative clusters (1–14) in each branch were then examined. All of the major clusters were found to be either functionally or structurally significant (Table 2). Some clusters in particular were found to have an important internal structural relationship and are discussed in detail below. Even though the *k-modes* algorithm is blind to the position of a site in the sequence, most of the discovered clusters contain sites that are either consecutive, or very close in sequence proximity, which one could expect to be structurally associated. This result suggests that the method presented here is able to pick up local structural associations remarkably well. Furthermore, there are some cases where the associated sites of the clusters are located at a distance (e.g., sites 30, 43, 68, and 69) with binding site relationships, or other structural or functional relationships (Table 2).

In addition to the Types I and II branches describing how the sites are interlaced, at least three types of attribute clusters were also observed which we designate: H-bonded clusters, van der Waals clusters, and extended clusters. *H-bonded clusters* contain residues that are in mutual H-bond contact. An example of this type of cluster is (1) shown in Figure 3B, forming a loop stabilized by no less than six predicted (PyMol) internal H-bonds. The second type of cluster, the *van der Waals cluster*, has sites that are not bonded but are in van der Waals contact. An example of this type is cluster (12 s), modeled in Figure 3C. The 1UBQ model reveals that the last cluster in the primary branch (12) (although quite dispersed along the primary sequence) contains van der Waals interactions, as well as predicted H-bonds, with sites 43 and 44 in van der Waals contact with sites 68 and 69, and two additional hydrogen bonds predicted by PyMol between site 44 and site 68. Hence, the clusters help to identify sites that are closely related in the tertiary structure, and often in actual contact, though widely separated in the primary sequence.

These first two types of clusters predicted by the pattern recognition software used in this research can readily be corroborated by mapping the attribute clusters to a solved 3D structure for the protein. The third type, however, designated as the *extended cluster*, is composed of sites that are distant both in the primary sequence, as well as the final 3D structure, yet they have a very high *SR*(*mode*) value, indicating strong association. When mapped onto the solved 3D structure of the protein, it is less obvious why the constituent sites form an attribute cluster. There are two possible reasons why some clusters may consist of sites that are not within van der Waals interaction distance from each other in the 3D structure, yet demonstrate a strong interdependency. First, it is possible that for extended clusters, the interdependence is due to the net effect of the conformation angles ψ and ϕ and the conformations of the side chains for the amino acids within the cluster [43] upon the short and medium range structure and free energy state of the protein in the area containing the extended attribute cluster. This net effect may be required to minimize free energy and directly maintain the stable tertiary structure. Second, the association of sites that are not structurally proximate to each other may be due to external functional constraints such as external binding as first conjectured by Wong et al. [9]. From Table 2, it can be observed that some clusters with no obvious internal structural relationship, such as 9, 11, 12, and 14, all contain external binding sites or, where the interaction is not yet known, external contact points with the external molecule it binds to [39]. Thus, the mutual interdependency of the sites in an extended cluster may be due to external binding requirements of functional controls where a number of sites are involved.

The success of this method in identifying important structural associations within ubiquitin does not mean that it will identify all such bonds, especially when the association among sites is weak. It does, however, demonstrate the ability to identify some key residue–residue contacts from the alignment data. This relationship among statistical association, molecular structure, and functionality is also consistent with earlier findings that identified the relationship among statistical patterns and 3D bonding structure [18] and functionality such as heredity factors in cancer suppressor genes [16,17]. The current results bring to light further details of statistical patterns that are strongly indicative of molecular structure and functionality in an alignment of proteins. Further work needs to be done to enable distinctions among clusters that predict residue-residue contacts, extended clusters that are keys in achieving structural stability, and those associated with external binding sites. One possible approach which has not been explored is to compare the FC of each site, which may be more heavily influenced by external binding, with the site clusters [6]. Another approach would be to compare the results of steered molecular dynamic computational modeling [44,45] on the more interesting parts of the cluster tree to see how those results would correlate with the cluster tree.

### The modules

**Figure 4:**
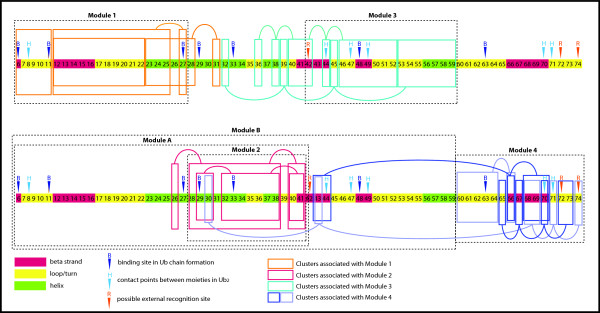
**Secondary structure of ubiquitin with locations of modules and major clusters.** The location of the major clusters associated with the four major modules is shown, for clarity, on two identical secondary structure ribbons, taken from model 1UBQ. An example of smaller clusters nested within larger clusters can be seen in the clusters associated with Module 2. The attribute (site) clusters associated with Modules 1 and 2 were found to be compact, with only a small region of interlacing involving sites 26, 28, and 31. Of particular interest are the two clusters associated with Module 4 that contain some sites that are quite distant in the primary structure. The key sites in these very extended clusters are sites 30, 43, and 69 which, although widely separated in the primary sequence, are actually in Van der Waals contact, as shown in Figure 3.

The final step was to examine the modules predicted by the cluster tree. For better visualization of the module locations and the major branch node-forming clusters on the sequence, Figure 4 shows the modules as boxes superimposed over the sequence with its color-coded secondary structure (taken from the 1UBQ model). Major clusters (color coded boxes) are also included. To avoid a confusing number of modules and clusters superimposed on just one sequence, two identical sequences are used for clarity, with two modules shown on each one.

To evaluate the validity of this modular view of ubiquitin, it has already been observed that at pH values of 6.8 to 7.5, the chemical shift perturbations are modular between the single ubiquitin molecule and the Lys-48 linked dimer form of ubiquitin (Ub_2_), with four areas of perturbations [41]. The same four areas of perturbations have also been observed for Lys-63 linked Ub_2_[40]. The remarkable correspondence among the four modules obtained by our method and the four areas of chemical shift perturbations is summarized in Table 4, providing validation that these four modules actually represent structural units within ubiquitin.

**Table 4:** Module and perturbation area comparison

**Module number**	**Sites spanned in module**	**Sites spanned in perturbation areas of Ub**_**2**_
1	6–25	6–20
2	26–41	20–39
3	42–59	40–60
4	60–74	61–76

#### Module 1

The tertiary structure of Module 1 is shown in Figure 3B. The cluster with the highest *SR*(*mode*) value, discovered in our experiment, was cluster (1), with a *SR*(*mode*) value of .26. Cluster (1) is also labeled in Figure 2 and consists of sites (7, 9–11). The high *SR*(*mode*) value shows that the sites are highly associated. From the 1UBQ model in Figure 3B, no less than six H-bonds are predicted by the MacPyMol model between Thr-7 and the other members of the cluster, sites 9–11. The number of internal H-bonds not only explains why it has such a high *SR*(*mode*) value, but it also indicates that site 7 is especially important within the cluster. Furthermore, the presence of the six predicted bonds in the cluster is an indication that cluster (1) provides structural stability to the loop and as a result, may have an important role in folding the two beta strands into a beta sheet. Site 11 within cluster (1) is also a known binding site [39] (Table 2).

Cluster (3) also has one of the higher *SR*(*mode*) values in the alignment, with a value of 0.19. From Figure 3B, it can be observed that this cluster, containing sites (18–19, 21) is part of the next loop after the beta sheet. In this particular cluster, there are two predicted bonds between Asp 21 and Glu 18, providing verification for the significance of the computationally discovered association. The double bond has the effect of providing structural rigidity to the loop.

#### Module 2

From Figure 2, the core of Module 2 is composed of three interlacing Type II branches 5 to 7, made up of attribute clusters from sites 29 to 43. An examination of the clusters for each branch reveals that the sites in each of the three clusters straddle sites in the other two. For this reason, these three branches appear to form a very stable, interlocking structural unit. The high internal structural stability predicted by the Type II branches may explain why, in earlier work by Varadan et al. [40,41], the area spanned by module 2 was observed to demonstrate significantly lower chemical shift perturbations than those observed in the areas spanned by the other three modules. It is often the case that sites within a cluster, though separated in the primary structure of sequence, may have members that are close once folding has occurred and may actually be bonded to each other. Sites 37 and 40 in cluster (7) are an example of this common occurrence. The 1UBQ model also provides a clear interpretation for cluster (5), by revealing the van der Waals relationship within the cluster between sites 30 and 41.

#### Module 3

The unique feature of this module stands out in the cluster tree (Figure 2) as it reveals its relative independence from the other modules. This leads to a prediction that the branches in module 3 may fold into a stable sub-domain first, and then the entire sub-domain folds into the already folded module A to form module B. It is also notable that module 3 has a very close correspondence to the previously discovered third perturbation area shown in Table 4[40,41].

#### Module 4

Recent work involving ubiquitin unfolding kinetics using hydrogen/deuterium exchange methods correlates with the results found here [46,47]. For example, the B5 strand, represented by module 4, appears to unfold before the loop and helix represented by primary branch 10 in module 3. This echoes what the cluster tree reveals regarding the independence of module 4. The cluster tree method demonstrated here may work very well in conjunction with unfolding studies in revealing details of protein folding.

### Transthyretin results

Transthyretin (TTR) is a homotetramer composed of a dimer of dimers. The tetramer contains a solvent channel within which is a binding pocket for two thyroxine molecules. Each dimer interface includes sites 114 to 120, forming an 8-strand, anti-parallel β-sheet, which constitutes the wall of the solvent channel. TTR is often found in complex with Retinol Binding Protein (RBP). At the center of this interface for human TTR are sites 20, 81, and 84 [48]. There are some TTR variants that result in amyloid formation. Two of the most common forms are Val30Met and Leu55Pro variants, both of which produce amyloidosis [49].

**Figure 5:**
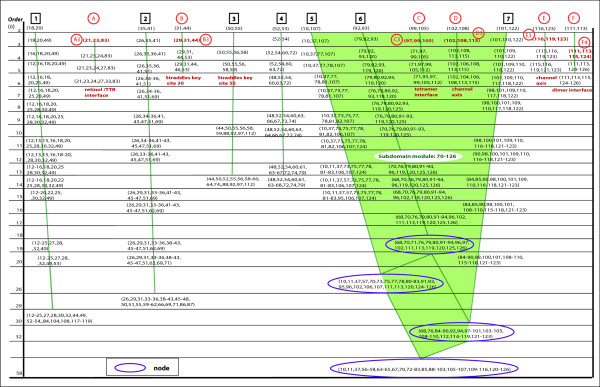
**TTR cluster tree.** The above diagram shows clusters of mutually associated sites found within a multiple sequence alignment for transthyretin, composed of 465 non-redundant sequences. It is remarkable that although some of the branches converge to a large node forming a major module, many of the branches do not converge to large nodes. This suggests that much of Transthyretin lacks the structural stability that comes with large nodes composed of many interdependent branches. This may provide insight as to why Transthyretin is susceptible to misfolding.

**Figure 6:**
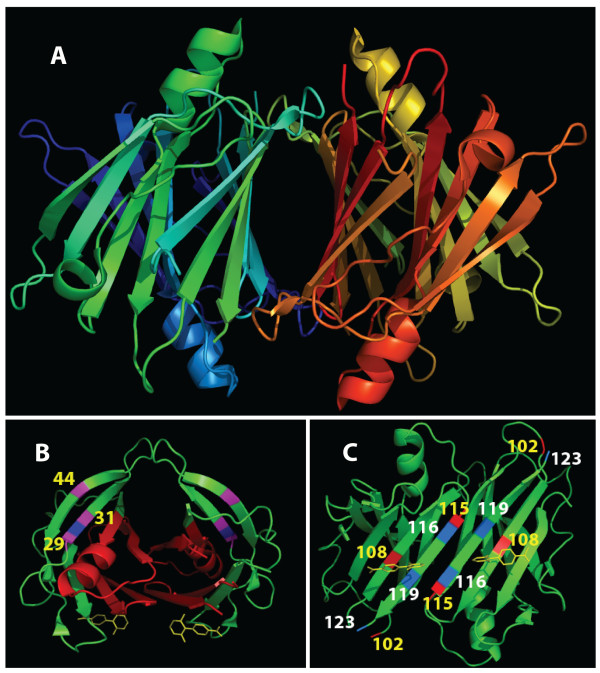
**Transthyretin subdomain module and clusters. (A)** The structure for the complete tetramer (pdb 1002), composed of four transthyretin monomers. **(B)** The sub-domain module, formed by three converging branches in the cluster tree (shown in light green in Figure 5) is shown in red. This module forms the dimer interface. Cluster B3 is shown in magenta in **(B)**. Its importance is indicated by the fact that it straddles site 30, for which there is a well known val30met mutation that leads to misfolding and amyloidosis. This suggests that cluster B3, consisting of sites 29, 30 and 44 **(B)**, has a critical roll in achieving a stable folded 3D structure. **(C)** Clusters D3 (labeled in yellow) and E3 (labeled in white) appear to be closely related. Each trio of mutually associated sites has remarkable symmetry, with one site at each end of the homotetramer channel, and one site in the center of the thyroxin binding site. The two residues at each end of both trios is likely important for the stabilization of the thyroxin pockets.

A multiple sequence alignment consisting of a total of 465 non-redundant sequences for the TTR family [50] was downloaded from Pfam and computationally analyzed using the *k*-modes algorithm. A large number of associated sites within the TTR alignment were discovered. The clusters were then arranged into a cluster tree as shown in Figure 5. The cluster tree for TTR shown in Figure 5 is remarkably different from the cluster tree for ubiquitin shown in Figure 2. Only three major branches, labeled as 5, 6, and 7 in Figure 5, converge into nodes forming a sub-domain module. This sub-domain module forms the TTR dimer interface and is shown in Figure 6B. Of the remaining, non-converging branches, there are six short branches that only achieve a fifth- or sixth-order cluster before terminating. These branches are labeled in Figure 5. Each of these branches are composed of hierarchically nested clusters, but only up to the fifth- or sixth-order. At that point, they abruptly end, rather than being absorbed into a higher order cluster or node. This suggests that the clusters in these branches play an important, but localized role in the structure or function of TTR. As mentioned earlier, each cluster within a nested hierarchical set is interesting to examine. To gain some understanding into these short branches, sample third-order clusters from branches A to F were examined individually within the context of the solved transthyretin structures available in the Protein Data Bank, pdb 2F7I, and pdb 1OO2 [51-54]. The importance of cluster A3 is revealed by its location, squarely at the TTR/RBP interface. This suggests that the clusters forming branch A may be critical to successful TTR/RBP binding. Cluster B3 straddles site 30, a site previously known to be highly sensitive to the proper folding of TTR, as mentioned above. Cluster B3 and site 30 are shown in Figure 6B. This suggests that cluster B3 is critical to the proper folding of TTR. The Val30Met variant may result in a failure in the formation of the β-sheet between sites 29 and 44 which, in turn, results in a misfolded structure prone to the formation of amyloids. Clusters D3 and E3, shown in Figure 6C, appear to be closely related in structure and function. Both trios consist of one site at either end of the channel β-sheet and one site at the centre, in close proximity to the thyroxin molecule. It appears that the two clusters play an important role in stabilizing that region of the β-sheet for the purpose of providing the proper structure for the thyroxin pocket. Cluster F4 is positioned at the dimer interface between the two TTR monomers. Cluster C3 is at the tetramer interface between the two dimers. All six clusters appear to be situated at key locations within the homotetramer that are either structurally important, or important for binding, or both.

The overall cluster tree for TTR is remarkably different from the ubiquitin cluster tree in that there are large branches in TTR that do not converge into the rest of the tree. This may suggest that TTR does not have the internal stability that ubiquitin has, and may be more prone to misfolding. Further work on other proteins that are prone to misfolding would be warranted here, to see if non-converging cluster trees are characteristic of potential instability. Those results would need to be compared with the cluster trees for strongly stable proteins. An overview of our results and predictions is provided in Table 3.

## Conclusions

We have introduced here a powerful new approach for analyzing the multiple sequence alignment data of protein families and discovering key associations among aligned sites and their importance within the 3D structure. Using two proteins of known structure and function as a test bench, our method revealed key associations among residues and sites that appear to have important structural and functional significance. It can, therefore, be applied to protein families of unknown structure and function. From our work, statistically analyzing the multiple aligned sequences for a protein family through the pattern discovery method presented in this paper revealed many key residue–residue contacts, as well as the sub-domain structure of ubiquitin and TTR. When the discovered attribute clusters were arranged according to their order, cluster trees were constructed which rendered further insights (dependent upon the hierarchy) into how the protein may fold. With our site clustering results, the secondary structure of ubiquitin forms four modules, which are closely associated with the four regions of perturbation in Lys-48 and Lys-63 linked Ub_2_ previously reported. Two categories of cluster branches, Types I and Type II, as we proposed, were discovered that may render a more a detailed understanding of how the protein folds. Furthermore, we observe that three types of attribute clusters were identified by our method, H-bonded, van der Waals, and extended clusters associated with binding sites. Such observations give additional insights into which associations of aligned sites make key contributions to a protein’s structural stability. Using the binding sites in the 1UBQ model and previous discoveries in the literature, we validate that the discovered clusters have both structural and functional significance. The TTR cluster diagram revealed further secrets of its 3D structure. Six short, non-converging branches were found, all of which contain clusters that have important structural or functional significance. Only one multi-branch sub-domain module was found for TTR, associated with the interface between the two monomers. The number of non-converging branches into large modules, however, suggests that TTR may be prone to instability when folding.

The method presented here, backed by previous work with ubiquitin and TTR, suggests that granular computing as a concept can make an important new framework for revealing the relationship between low-order (three or four residues) residue–residue contacts and the demarcation of higher-order sub-domains, using a cluster tree. This sub-molecular hierarchical view also identifies sites within a protein that may be of particular structural or functional importance in the design of new drugs, for example. The ability to discover key residue–residue contacts, branches, and larger structural sub-domains within a protein through the *k-*modes analysis of the multiple sequence alignment will be a significant asset in understanding the details in the sequence of protein folding, structure, and functionality among different residue locations within a hierarchical global protein framework. Furthermore, by discovering the important attribute clusters within a protein, predictions can also be made as to which mutations could be more harmful or more stable than others. All these play an important role in furthering our understanding of the information processing capability of genes and proteins, in terms of the specific use of functional units at specific locations on the sequence to create the 3D structure as well as the internal and external functionality of the molecules.

## Methods

The ubiquitin protein family was chosen as a suitable workbench to test our method for three reasons. First, the number of samples is reasonably large, numbering a few thousand, permitting a meaningful statistical analysis. Second, the structure of ubiquitin is well known and a 3D model is available from the Protein Database [38]. The available 1UBQ 3D model permitted us to compare the results and analyze their implication in terms of their structure and folding. Finally, there is considerable knowledge of the functions of ubiquitin, the most well known of which is the labeling of proteins for destruction within the cells of eukaryotic life [39,55].

In our experiment, an aligned set of 2,442 sequences for ubiquitin was downloaded from the Pfam database [56]. The data were then computationally post processed to retain columns less than 20% gapped. After the insertions and duplicate sequences were removed, the number of unique sequences remaining was 1,066 and the number of aligned sites remaining was 69. The alignment did not include the first 5 sites that were missing from the Pfam alignment at the time we performed our analyses. These 69 sites represented sites 6–74 of the ubiquitin primary structure using the 1UBQ 3Dmodel from the protein data base [38].

At the onset of our experiment, the pre-processed data, consisting of 1,066 aligned, unique sequences was then analyzed using the *k*-modes algorithm for discovering clusters of attributes (sites) within the primary structure of ubiquitin. The data were also analyzed to compute the *SR*(*mode*) value for each cluster.

The first step in analyzing the results was to manually arrange the clusters into a cluster tree as described earlier in the Section “Attribute clusters and information granules”. Branches containing the lowest-order clusters are denoted as *primary branches* and are numbered from 1 to 14 across the top of Figure 2. Within a nested hierarchical set of clusters, each cluster will be informative to examine; there is no official representative cluster. One way of choosing a representative cluster is to pick out clusters that have higher mutual interdependency than others, indicated by a high *SR*(*mode*) for the cluster. For the ubiquitin family only, this method was chosen, although any other cluster could have been picked as a representative cluster for a particular branch. The first step was to compute the *SR*(*mode*) value for each cluster in the primary branch. The cluster in each branch with the highest *SR*(*mode*) value was chosen as the representative cluster, since it had the highest mutual interdependency. If two clusters in the same branch had similar values for *SR*(*mode*) the cluster with the higher order was selected, since the higher-order cluster would contain more sites and, thus, be more informative and interesting. For the same reason, if a higher-order cluster in the branch had a *SR*(*mode*) value that was only slightly lower than the highest *SR*(*mode*) value of another, lower-order cluster, the higher-order cluster instead was chosen as the representative cluster for that primary branch. For consistency, a certain percentage difference can be chosen as the cutoff. These representative clusters, each composed of two or more sites with a high interdependence as indicated by the *SR*(*mode*) value, were then computationally analyzed to determine what patterns (associations of specific amino acids within the sites spanned by the attribute cluster) were contained within each representative cluster. The residual of each of the patterns was also computed. The discovered attribute clusters of ubiquitin were examined in terms of their relevance to the 3D structure using the 1UBQ model. They were also analyzed for function as described in the literature. Statistically significant patterns were computationally discovered by searching for amino acid combinations that had an adjusted residual value greater than 3.29, which corresponds to a confidence level greater than 99.9%. A multiple sequence alignment for TTR, downloaded from Pfam [50], was also analyzed using the same procedure outlined above for ubiquitin with the exception as to how the representative clusters were chosen. Since all clusters are statistically significant, any cluster can be chosen as a sample cluster. For TTR, we chose the third-order clusters as the sample clusters. Since the criterion was different for TTR, they were referred to as sample clusters rather than representative clusters. Ideally, if time and resources permit, all clusters should be examined to more fully understand their significance.

## Abbreviations

3D: Three-dimensional; DNA: Deoxyribonucleic acid; ETM: Evolutionary trace method; FC: Functional complexity; *G*: The set of 20 normally occurring amino acids; *K*: Number of clusters to be output by the *k*-modes algorithm; *M*: Number of sequences in a multiple sequence alignment; *N*: Number of sites in an individual protein or multiple sequence alignment; NMR: Nuclear magnetic resonance; RBP: Retinol binding protein; *Rii'*: Normalized redundancy measure; RNA: Ribonucleic acid; *SR*(*i*): Normalized interdependency redundancy; TTR: Transthyretin; Ub_2_: Ubiquitin dimer; *X*: A *N*-tuple; *Y*: Represents a cluster of mutually associated sites; α: Represents a particular amino acid; λ: Represents a pattern of amino acids.

## Competing interests

The authors declare that they have no competing interests.
